# Patient characteristics, treatment patterns, and outcomes of hormone receptor-positive, human epidermal growth factor receptor 2-negative advanced breast cancer patients prescribed cyclin-dependent kinase 4 and 6 inhibitors: large-scale data analysis using a Japanese claims database

**DOI:** 10.1007/s10549-022-06816-9

**Published:** 2022-11-21

**Authors:** Masaaki Kawai, Masahiro Takada, Takahiro Nakayama, Norikazu Masuda, Hirokazu Shiheido, Zhihong Cai, Yu-Jing Huang, Tsutomu Kawaguchi, Yoshinori Tanizawa

**Affiliations:** 1 Department of Surgery I, Yamagata University Graduate School of Medical Science, Yamagata, Japan; 2 Department of Breast Surgery, Kyoto University Graduate School of Medicine, Kyoto, Japan; 3 Department of Breast and Endocrine Surgery, Osaka International Cancer Institute, Osaka, Japan; 4 Department of Breast and Endocrine Surgery, Nagoya University Graduate School of Medicine, Nagoya, Japan; 5 Japan Drug Development and Medical Affairs, Eli Lilly Japan K.K., Kobe, Japan; 6 Global Patient Safety, Eli Lilly and Company, Indianapolis, IN USA

**Keywords:** CDK4 and 6 inhibitor, Administrative claims database, Effectiveness, Metastatic breast cancer, Real-world evidence, Safety

## Abstract

**Purpose:**

The aim was to understand real-world cyclin-dependent kinase (CDK) 4 and 6 inhibitor use in Japan.

**Methods:**

This retrospective observational study used a Japanese administrative claims database and included patients with presumptive hormone receptor-positive, human epidermal growth factor receptor 2-negative advanced breast cancer (ABC) prescribed CDK4 and 6 inhibitor therapy between December 2017 and March 2021. Patient characteristics, treatment patterns, and selected clinical and safety outcomes were descriptively summarized. Time to discontinuation (TTD) and chemotherapy-free survival (CFS) were examined using Kaplan–Meier estimates.

**Results:**

The study cohort (*N*** = **6442) was predominantly female (99.4%; median [range] age 64 [26–99] years) with records of metastases (79.6%) within 1 year prior to initiating CDK4 and 6 inhibitor therapy. In total, 4463 (69.3%) and 1979 (30.7%) were prescribed palbociclib and abemaciclib, respectively, as their first CDK4 and 6 inhibitor, most commonly in combination with fulvestrant (*n* = 3801; 59.0%). Overall, 3756 patients initiated a subsequent anticancer treatment, of whom 748 (19.9%) initiated a different CDK4 and 6 inhibitor in combination with the same or different endocrine therapy. Median TTD (95% confidence interval) was 9.7 (9.3, 10.1) months for the first CDK4 and 6 inhibitor therapy. Median CFS was 26.1 (24.6, 27.8) months. Incidence of clinically relevant diarrhea was higher after abemaciclib initiation (9.8%) than after palbociclib initiation (1.5%). More patients experienced dose reduction with palbociclib (69.3%) than with abemaciclib (53.0%).

**Conclusion:**

The data provide insights into current clinical practices for CDK4 and 6 inhibitor use in Japan that could help establish future treatment strategies for ABC.

**Supplementary Information:**

The online version contains supplementary material available at 10.1007/s10549-022-06816-9.

## Introduction

Breast cancer represented 21.4% of new cancer diagnoses in Japanese women in 2020 [1]. More than two-thirds of breast cancers are hormone receptor-positive (HR+), human epidermal growth factor receptor 2-negative (HER2−) [2]. Until recently, front-line therapy for this type of cancer was endocrine therapy (ET), but resistance to ET is a common issue for patients with advanced breast cancer. Cyclin-dependent kinase (CDK) 4 and 6 are targets for anticancer therapy based on their roles in regulating cellular proliferation [3]. Three selective CDK4 and 6 inhibitors, palbociclib, ribociclib, and abemaciclib, have been developed for clinical use in HR+, HER2− breast cancer. All 3 treatments have been shown to significantly improve progression-free survival (PFS) in HR+, HER2− advanced breast cancer when combined with ET in the first or second line [4–12], with ribociclib and abemaciclib also shown to significantly improve overall survival (OS) in these settings [13–17].

CDK4 and 6 inhibitors are still a relatively new class of drugs in Japan. Among the three drugs, palbociclib and abemaciclib have been approved for clinical use in Japan, with palbociclib receiving its first regulatory approval for unresectable or recurrent breast cancer in 2017 and abemaciclib receiving approval 1 year later for advanced HR+, HER2− breast cancer [18]. Palbociclib and abemaciclib have become extensively used in Japan, with CDK4 and 6 inhibitor/ET combination therapy now the standard of care for advanced HR+, HER2− breast cancer [19]. Subpopulation analyses of clinical trials have demonstrated the efficacy and safety of palbociclib and abemaciclib in Japanese patients [20–23]. However, findings from clinical trials can be poorly generalizable to all patient populations and treatment situations, largely due to extensive eligibility criteria for patient enrollment. Real-world databases collect data from larger, more heterogeneous populations of patients and therefore can provide insights beyond those of clinical trials.

Given the widespread use of CDK4 and 6 inhibitors in Japan, a better understanding of their effectiveness and safety in real-world clinical practice is important to assess the benefit-risk of these treatments. The primary objective of this study was to describe the demographics and characteristics, treatment patterns, and clinical outcomes of Japanese patients with advanced HR+, HER2− breast cancer who were prescribed CDK4 and 6 inhibitors, using a large claims-based database. The secondary objectives were to describe the occurrence of adverse events (AEs) of interest, utilization of concomitant medications, and monitoring tests during CDK4 and 6 inhibitor treatment.

## Materials and methods

### Study overview

This was a retrospective observational study that used the Medical Data Vision Co., Ltd. (MDV) database, a de-identified database of discharge summaries and health insurance claims from hospitalizations and outpatient visits at Japanese hospitals that use the Diagnosis Procedure Combination (DPC) system [24]. The DPC system includes hospitals that provide acute phase medical care and other services and includes most high-volume medical and cancer centers in Japan. As of December 2021, the MDV database contained data of approximately 38 million patients from more than 458 hospitals, representing 26% of DPC hospitals in Japan [25].

This study was conducted in accordance with the Declaration of Helsinki and Good Pharmacoepidemiology Practices. Per the Japanese Ethical Guidelines for Medical and Health Research Involving Human Subjects [26], ethical review and informed consent were not required, as this was a noninterventional, retrospective study that used anonymized patient data.

### Study design and cohorts

**Fig. 1:**
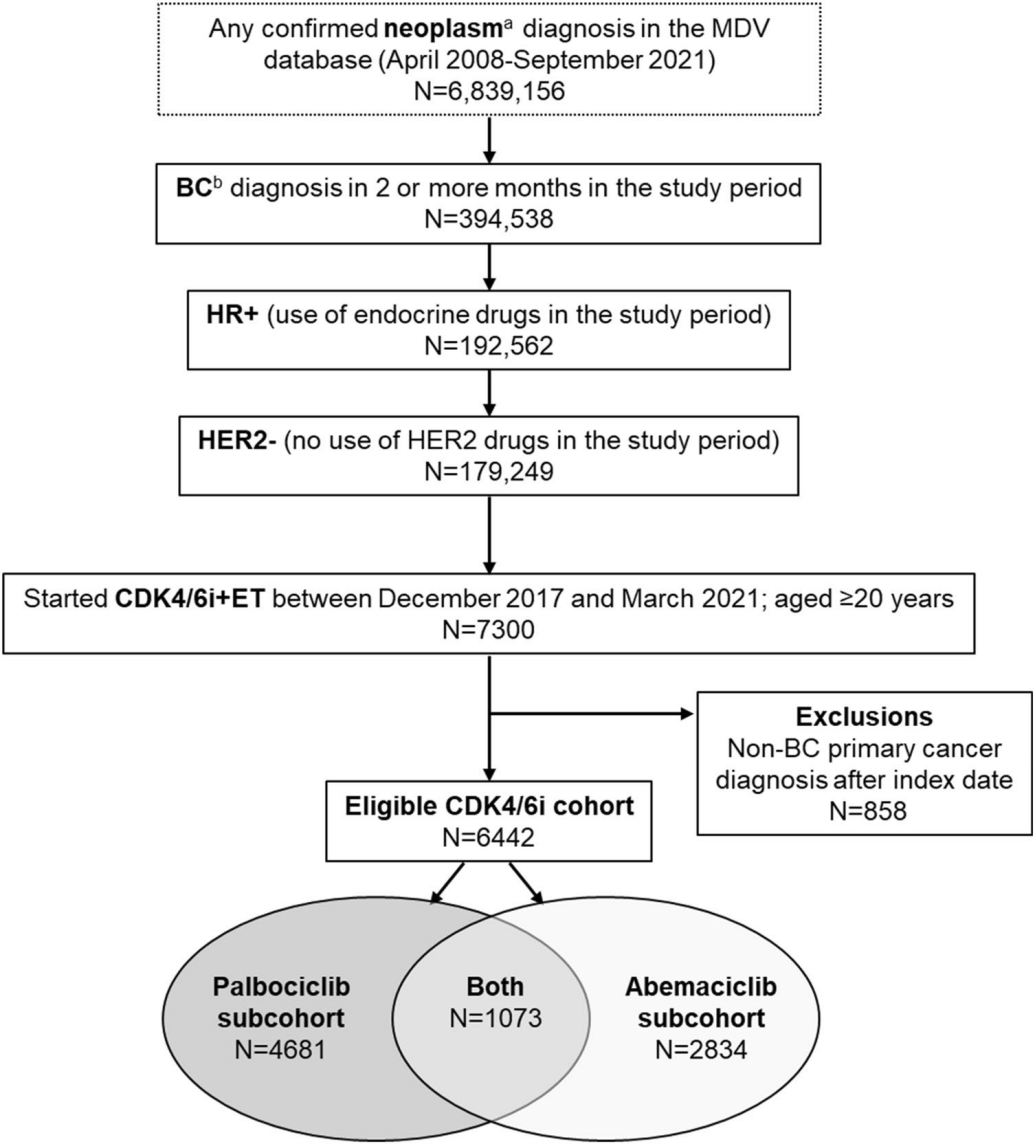
Patient selection. The data source for this study was the MDV database, a hospital-sourced anonymized claims database in Japan. The study cohort consisted of patients with presumptive HR + , HER2− advanced breast cancer who were prescribed CDK4 and 6 inhibitors within a specified identification period (December 2017 to March 2021). The index date was defined as the date of the first prescription of CDK4 and 6 inhibitors [abemaciclib or palbociclib] during the study period (December 2012 to September 2021). *BC* breast cancer, *CDK4/6i* cyclin-dependent kinase 4 and 6 inhibitor; *ET* endocrine therapy, *HER2−* human epidermal growth factor receptor-2-negative, *HR* + hormone receptor-positive, *ICD-10* International Statistical Classification of Diseases and Related Health Problems, 10th revision, *MDV* Medical Data Vision. **a** ICD-10 [27], diagnostic codes C00-D48. **b** ICD-10 [27], diagnostic codes C50

The study design comprised a study period from December 2012 to September 2021 (5 years prior to first CDK4 and 6 inhibitor availability in Japan to end of data availability), an index date (date of the first prescription of CDK4 and 6 inhibitors during the study period), and a baseline period (up to 365 days prior to the index date, unless otherwise specified). This study used data from a pool of 6,839,156 patients who had at least one confirmed diagnosis of neoplasm (International Statistical Classification of Diseases and Related Health Problems, 10^th^ revision [ICD-10] [27]; codes C00-D48; Figure 1) in the MDV database between April 2008 (start of database data collection) and September 2021. From this starting population, the overall study cohort, comprising breast cancer patients who were prescribed CDK4 and 6 inhibitors (termed the “CDK4/6i cohort”), was defined based on the following criteria: (1) confirmed diagnosis codes of breast cancer (C50 except breast sarcoma) in at least two different months; (2) prescription of ET (surrogate for HR+); (3) no prescription of anti-HER2 drugs (surrogate for HER2−); (4) index date between December 2017 and March 2021; and 5) age ≥20 years at the index date. ET was required to be prescribed within 21 days from the index date. Patients were excluded from the study if they had any confirmed diagnosis of primary cancer other than breast cancer from the index date to the end of the follow-up period. Patients who had any prescription of abemaciclib or palbociclib during follow-up were assigned to the “abemaciclib” or “palbociclib” subcohort, respectively, and could be included in both subcohorts if they were subsequently prescribed another CDK4 and 6 inhibitor during the follow-up period.

### Patient and hospital characteristics

Patient characteristics and hospital information were summarized during the baseline period. Weight and height information (used to calculate body mass index [BMI]), smoking history, and the results of the 10-item Barthel Activities of Daily Living (ADL) index [28] were obtained from discharge summaries from the last hospital admission in the baseline period (if any). Patient treatment history was summarized for up to 5 years preceding the index date. Prescription of luteinizing hormone-releasing hormone (LHRH) during the follow-up period was summarized as surrogate for premenopausal status.

### Treatment patterns and duration of therapy

For the first CDK4 and 6 inhibitor therapy, the patterns of treatment with breast cancer drugs subsequently prescribed during the follow-up period were summarized by Sankey plot. The breast cancer drugs examined (Online Resource 1) correspond to anticancer drugs and ETs in the Japanese guidelines for systemic breast cancer treatment [19].Treatment regimens were defined as the combination of breast cancer drugs prescribed within the first 21 days of each line of therapy. Lines of therapy were considered ended when all the breast cancer drugs in the regimen were terminated or a new breast cancer drug was added to the regimen, whichever occurred first. Duration of therapy was evaluated using time to discontinuation (TTD), defined as the time from the prescription of the first agent in the regimen to the discontinuation of the last agent in the regimen. Patients were considered to be continuing the line and were censored at the last administration date of the line if there were ≤90 days between the end of line and end of data without a subsequent line of therapy. Chemotherapy-free survival (CFS) and intravenous CFS (defined as the duration from the index date to the earliest use of any chemotherapy or intravenous chemotherapy, respectively, or death) were also estimated. Drugs included in these analyses are indicated in Online Resource 1. Patients without chemotherapy were censored at the last hospital visit. Only patients without chemotherapy in the 1-year baseline period were included in the analysis.

To explore therapy duration when CDK4 and 6 inhibitors were used in relatively early lines of therapy, TTD and CFS were also determined after removing patients prescribed breast cancer drugs before the index date that are indicated only for metastatic breast cancer in Japan. For this analysis, metastatic breast cancer drugs included capecitabine, gemcitabine, tegafur/gimeracil/oteracil, irinotecan, vinorelbine, nab-paclitaxel, bevacizumab, eribulin, everolimus, palbociclib, abemaciclib, and olaparib, but not fulvestrant, which may be used in earlier lines of therapy.

### Outcomes from subcohort analyses

Each “palbociclib period” or “abemaciclib period” was defined as the period from first prescription to the last projected dose date (Online Resource 2). The initial prescription dosage (mg/day) was evaluated on the first day of each abemaciclib or palbociclib period. For patients who continued CDK4 and 6 inhibitor therapy for ≥30 days prior to discontinuing therapy, dose reduction and medication possession ratio (MPR) of CDK4 and 6 inhibitors, utilization of concomitant medications, monitoring tests, and AEs of interest were evaluated for the abemaciclib and palbociclib periods.

Selected events of neutropenia, venous thromboembolism (VTE), liver disease, and diarrhea were defined as AEs of interest in this study. Only AEs of interest that occurred during the palbociclib/abemaciclib treatment period but not during a specified baseline period prior to the index date for each AE were summarized (30 days for diarrhea and neutropenia, 60 days for liver disease, and 365 days for VTE). Neutropenia events of interest were identified based on a diagnosis of neutropenia plus prescription of ≥1 granulocyte colony stimulating factor (G-CSF) drugs in the same claim month. VTEs of interest were identified based on a previous published claims-based algorithm [29]. Liver AEs of interest were identified based on a diagnosis of liver disease or the prescription of liver protection drugs and were excluded if liver cancer or liver metastasis was detected within ±1 month of the claimed event. Diarrhea events of interest were identified based on diagnoses of diarrhea and dehydration or abnormal electrolyte levels in the same claim month.

### Statistical analysis

Descriptive statistics are presented as *n* (%), mean with standard deviation, and/or median (minimum-maximum or interquartile range [IQR]), as applicable. No statistical comparisons between groups were planned, therefore no statistical adjustments for bias or confounding factors were conducted. Missing data were not imputed. Subcohort data were summarized for the first therapy with each of abemaciclib and palbociclib, regardless of prior CDK4 and 6 inhibitor use.

The Kaplan-Meier method was used to estimate medians and 95% confidence intervals (CIs) for TTD and CFS. TTD was evaluated in the CDK4/6i cohort for the first CDK4 and 6 inhibitor, first subsequent therapy, and all breast cancer drugs (from the index date). TTD was evaluated in the subcohorts for the first abemaciclib or palbociclib use and by presence or absence of prior CDK4 and 6 inhibitor use.

## Results

### Patient selection

Figure 1 represents study cohort construction. A total of 6442 patients were identified for the CDK4/6i cohort. The palbociclib and abemaciclib subcohorts comprised 72.7% (4681/6442) and 44.0% (2834/6442) of the patients, respectively, including 1073 patients who were prescribed both palbociclib and abemaciclib during the follow-up period and were therefore included in both subcohorts.

### Patient and hospital characteristics

The majority of patients were prescribed CDK4 and 6 inhibitors at a designated cancer hospital (85.6%) within a surgery-related department (94.4%; Table 1). Most patients were female (99.4%), with a median age of 64 years (range: 26-99 years; age distribution shown in Online Resource 3). Among 909 (14.1%) patients who were prescribed LHRH agonists, 689 (10.7%) and 220 (3.4%) were aged <50 years and ≥ 50 years, respectively. The majority had records of metastases during the baseline period (79.6%), most commonly in bone (52.3%) or visceral organs (43.3%). During the treatment history period, 77.2% of patients had received ET, most frequently fulvestrant (37.2%) or nonsteroidal aromatase inhibitors (letrozole, 32.1%; anastrozole, 21.0%), and 40.7%, 20.2%, and 16.4% had received anticancer drugs, radiotherapy, and breast cancer surgery, respectively (Table 1). Treatment history is detailed in Online Resource 4.

**Table 1:** Hospital and patient characteristics of the CDK4/6i cohort

*n* (%)^a^	CDK4/6i cohort *N* = 6442
Hospital characteristics	
Number of beds	
< 200	119 (1.9)
200–499	2997 (46.5)
≥ 500	3326 (51.6)
Designated cancer hospital	5514 (85.6)
Department	
Surgery	6082 (94.4)
Internal medicine	348 (5.4)
Other or unknown	12 (0.2)
Patient characteristics	
Age (years) at index date	
Median	64
Minimum–maximum	26–99
Sex	
Female	6400 (99.4)
Male	42 (0.7)
LHRH in the follow-up period^b^	909 (14.1)
Metastases in the 1-year baseline period, any^c^	5130 (79.6)
Visceral	2786 (43.3)
Lung	1467 (22.8)
Liver	1176 (18.3)
Brain	328 (5.1)
Bone	3370 (52.3)
Data available during hospitalization	
Patients with hospitalization during the baseline period	1656 (25.7)
Days between last hospital admission during the baseline period and index date, median (IQR)	88 (27–204)
Weight^d^	
Record available from last hospital admission during the baseline period	1632 (25.3)
Mean (SD), kg	55.1 (11.0)
BMI^d^	
Record available from last hospital admission during the baseline period	1625 (25.2)
Mean (SD), kg/m^2^	23.1 (4.4)
Smoking history^d^	
Record available from last hospital admission during the baseline period	1552 (24.1)
Yes	227 (14.6)
No	1325 (85.4)
Total ADL independence^e^	
Record available from last hospital admission during the baseline period	1564 (24.3)
Dependent	392 (25.1)
Independent	1172 (74.9)
Treatment history^f^	
Breast cancer surgery	1056 (16.4)
Months between first breast cancer surgery and index date	
Median (IQR)	32.1 (18.6–45.7)
Radiotherapy	1303 (20.2)
Months between first radiotherapy and index date	
Median (IQR)	21.5 (7.5–39.3)
Endocrine therapy	4970 (77.2)
Months between first endocrine therapy and index date	
Median (IQR)	31.0 (12.6–54.2)
Anticancer drugs	2619 (40.7)
Months between first anticancer drugs and index date	
Median (IQR)	25.8 (13.2–43.0)

^a^*n* (%) shown where % was calculated using the number of patients in the study cohort as the denominator, unless otherwise indicated below

^b^Prescription of LHRH during the follow-up period (from the index date to the end of follow-up) was surrogate for menopausal status (patients presumed to be premenopausal)

^c^Based on the presence of one or more ICD-10 codes of metastasis (C77-79) in the database. Visceral metastases were defined by claim codes C78-C79 except for C79.2/5/9 and metastasis in the breast included in C79.8

^d^For subcategories, percentages were calculated using the number of patients with data recorded from last hospital admission during the baseline period as the denominator

^e^As assessed on the 10-item Barthel ADL index at hospital admission. Total ADL was defined as “independent” if all 10 items were recorded as independent and “not independent” if any items were recorded as not independent. For patients with any ADL items unknown or missing, ADL was defined as “missing”. For subcategories, percentages were calculated using the number of patients with ADL data recorded from last hospital admission during the baseline period as the denominator

^f^Treatment history for a period of up to 5 years prior to the index date

*ADL* activities of daily living, *BMI* body mass index, *CDK* cyclin-dependent kinase, *CDK4/6i* CDK4 and 6 inhibitor, *ICD-10* international statistical classification of diseases and related health problems, 10th revision, *IQR* interquartile range, *LHRH* luteinizing hormone-releasing hormone, *SD* standard deviation

### Treatment patterns

**Fig. 2:**
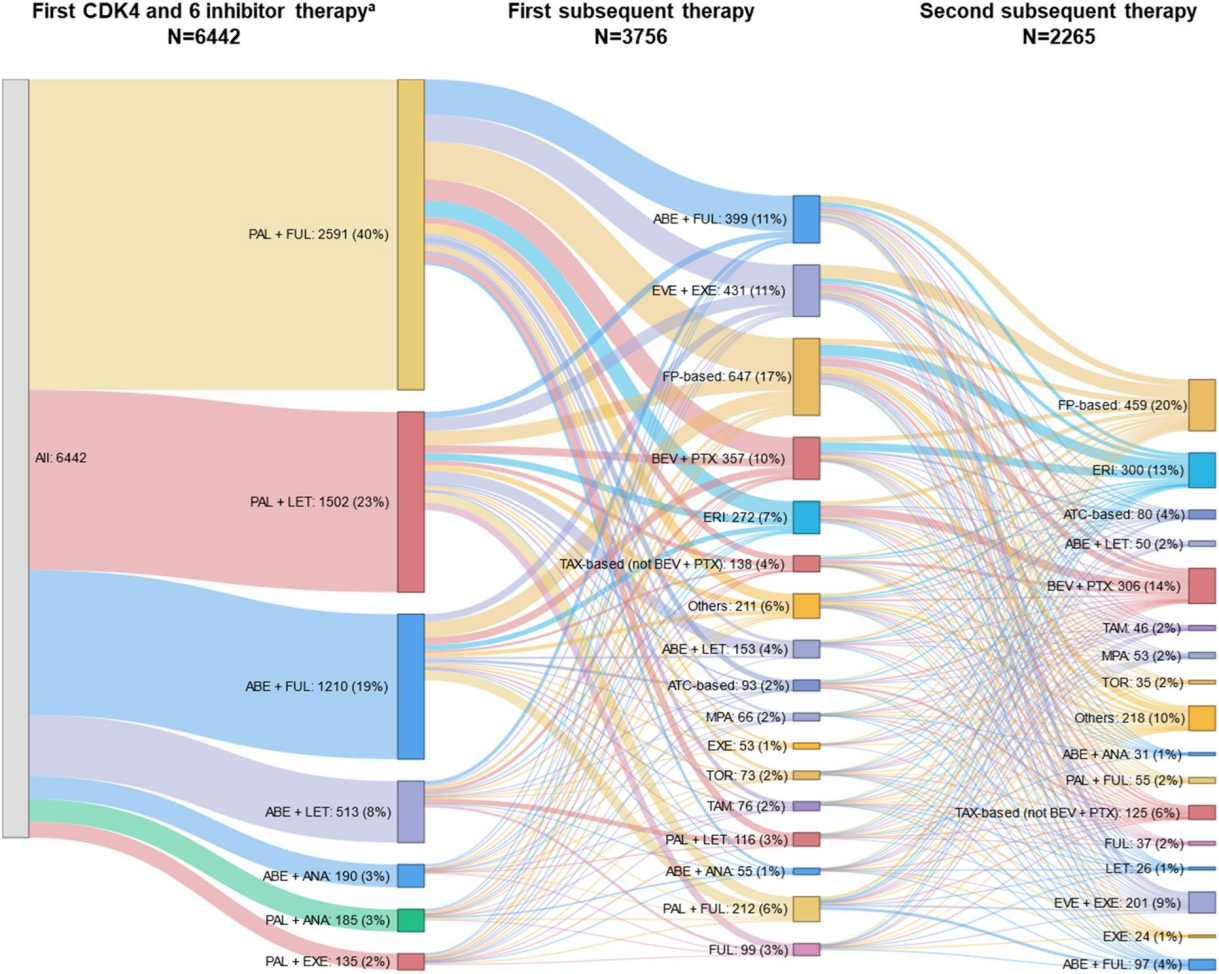
Treatment pattern. Sankey chart showing treatments in > 1% of patients for the first CDK4 and 6 inhibitor therapy and the 2 subsequent lines of therapy (if any). *n* (%) shown wherein *N* for each regimen includes patients who have not discontinued the line of therapy. **a** This study describes the treatment pattern only after starting CDK4 and 6 inhibitors. Therefore, the first CDK4 and 6 inhibitor therapy in this study may not be the first systemic therapy (first-line) for metastatic breast cancer. *ABE* abemaciclib, *ATC* anthracycline, *ANA* anastrozole, *BEV* bevacizumab, *CDK* cyclin-dependent kinase, *ERI* eribulin, *ET* endocrine therapy, *EVE* everolimus, *EXE* exemestane, *FP* fluoropyrimidine, *FUL* fulvestrant, *LET* letrozole, *MPA* medroxyprogesterone acetate, *PAL* palbociclib, *PTX* paclitaxel, *TAM* tamoxifen, *TAX* taxane, *TOR* toremifene

Overall, 4463 (69.3%) and 1979 (30.7%) patients were prescribed palbociclib and abemaciclib, respectively, as their first CDK4 and 6 inhibitor therapy. In 2017 and 2018 (when only palbociclib was approved in Japan), almost 100% of the CDK4/6i cohort were prescribed palbociclib as their first CDK4 and 6 inhibitor. After abemaciclib approval, the proportion of patients prescribed abemaciclib as their first CDK4 and 6 inhibitor increased to approximately half of the population in 2020 and 2021 (Table 2). The most common initial CDK4 and 6 inhibitor regimen was combination therapy with fulvestrant (n=3801; 59.0%; Figure 2).

**Table 2:** Proportion of patients prescribed their first CDK4 and 6 inhibitor by starting year (CDK4/6i cohort)

Treatment, *n* (%)	Overall	2017	2018	2019	2020	2021
Palbociclib	4463 (69.3)	75 (100.0)	1762 (98.7)	1201 (62.1)	1134 (53.7)	291 (54.5)
Abemaciclib	1979 (30.7)	0	24 (1.3)	734 (37.9)	978 (46.3)	243 (45.5)
Total	6442	75	1786	1935	2112	534

*CDK* cyclin-dependent kinase, *CDK4/6i* CDK4 and 6 inhibitor

Following the first CDK4 and 6 inhibitor therapy, 3756 (58.3%) patients initiated a subsequent regimen. Patient flow from the first CDK4 and 6 inhibitor therapy to the first and second subsequent therapies is shown in Figure 2. The highest proportion of patients (19.9%) initiated a different CDK4 and 6 inhibitor in combination with the same or different ET, and 10.8% maintained the same CDK4 and 6 inhibitor but in combination with a different ET (Table 3). Other common (>10%) subsequent therapies included fluoropyrimidine-based chemotherapy (17.6%), ET monotherapy (11.5%), and everolimus plus exemestane (11.5%; Table 3).

**Table 3:** First subsequent therapies prescribed after the first CDK4 and 6 inhibitor therapy (CDK4/6i cohort)

First subsequent therapy, *n* (%)	CDK4/6i cohort *N* = 6442
Patients who initiated a subsequent therapy	3756 (58.3)
CDK4 and 6 inhibitor + ET (CDK4 and 6 inhibitor changed)^a^	748 (19.9)
FP-based	659 (17.6)
ET monotherapy (another ET)	432 (11.5)
EVE + EXE	431 (11.5)
CDK4 and 6 inhibitor + ET (ET changed)	407 (10.8)
BEV + PTX	359 (9.6)
ERI	272 (7.2)
Others	216 (5.8)
Taxane-based (not BEV + PTX)	138 (3.7)
ATC-based	94 (2.5)

^a^Category includes patients prescribed CDK4 and 6 inhibitor/ET combination regimens who subsequently either initiated a different CDK4 and 6 inhibitor with the same ET or both a different CDK4 and 6 inhibitor and a different ET

*ATC* anthracycline, *BEV* bevacizumab, *CDK* cyclin-dependent kinase, *CDK4/6i* CDK4 and 6 inhibitor, *ERI* eribulin, *ET* endocrine therapy, *EVE* everolimus, *EXE* exemestane, *FP* fluoropyrimidine, *PTX* paclitaxel

The median TTD was 9.7 months (95% CI: 9.3, 10.1) for the first CDK4 and 6 inhibitor therapy (Table 4). Median TTD for the first subsequent therapy was numerically longest for regimens containing targeted agents, including bevacizumab (6.0 months), everolimus (5.0 months), and CDK4 and 6 inhibitor regimens (5.5-8.5 months; Online Resource 5). Median TTD for all breast cancer drugs was 36.0 months (95% CI: 34.5, 37.9) from the index date, whereas median CFS was 26.1 months (95% CI: 24.6, 27.8) and median intravenous CFS was 35.2 months (95% CI: 33.0, 37.7; Table 4). To investigate treatment duration when CDK4 and 6 inhibitors were used in early lines of therapy, analyses conducted after removing patients who received drugs for metastatic breast cancer before the index date showed the types of first subsequent therapies in this group were similar to those of the entire CDK4/6i cohort (Online Resource 6), but TTDs were longer (Online Resource 7).

**Table 4:** Time to discontinuation of therapy and chemotherapy-free survival (CDK4/6i cohort)

Time-to-event measure	Patients (*N*)	Events (*n*)	Median (months)	95% CIs (months)
TTD, first CDK4 and 6 inhibitor therapy^a^	6442	4539	9.7	9.3, 10.1
TTD, first subsequent therapy^a^	3756	2845	5.5	5.3, 5.8
TTD, overall breast cancer drugs (from index date)^b^	6442	2351	36.0	34.5, 37.9
CFS^c^	4650	1873	26.1	24.6, 27.8
Intravenous CFS^d^	4650	1495	35.2	33.0, 37.7

Treatment regimens were defined as the combination of breast cancer drugs that were prescribed within the first 21 days of each line of therapy. The line of therapy ended when the patient either: 1) terminated all the breast cancer drugs in the regimen (end date: date of last prescription plus the number of days of supply -1 day); or 2) added a new breast cancer drug that was not included in the regimen (i.e., causing the treatment line to advance), whichever occurred first

^a^Patients were considered to be continuing the line and were censored at the last administration date of the line if there were ≤ 90 days between the end of the line and the end of data without a subsequent line of therapy

^b^Patients with ≤ 90 days between the estimated last dose of breast cancer drugs and end of data were censored for therapy duration at the last dose, as such patients were likely to be on treatment at the last visit

^c^Time from the CDK4 and 6 inhibitor index date to the date of first chemotherapy use or death. If no events occurred, patients were censored at the last hospital visit record. Only patients without chemotherapy in the 1-year baseline period were included in the analysis

^d^Time from the CDK4 and 6 inhibitor index date to the date of first intravenous chemotherapy use or death. If no events occurred, patients were censored at the last hospital visit record. Only patients without chemotherapy in the 1-year baseline period were included in the analysis

*CDK* cyclin-dependent kinase, *CDK4/6i* CDK4 and 6 inhibitor, *CFS* chemotherapy-free survival, *CI* confidence interval, *TTD* time to discontinuation

### Treatment duration and other clinical outcomes in the subcohorts

Median TTD of the first therapy with abemaciclib and palbociclib in the subcohorts, regardless of prior CDK4 and 6 inhibitor use, was 9.3 (95% CI: 8.7, 10.0) months for abemaciclib and 8.7 (95% CI: 8.1, 9.0) months for palbociclib (Table 5). The duration of therapy was numerically shorter in patients who were prescribed another CDK4 and 6 inhibitor therapy previously (median: 7.0–7.3 months) than in patients who had not (median: 8.8-10.5 months; Table 5). Combination treatment with fulvestrant was the most common regimen prescribed in both the abemaciclib and palbociclib subcohorts (approximately 58%–59%).

**Table 5:** Duration of first abemaciclib/palbociclib therapy by presence of prior CDK4 and 6 inhibitor use (abemaciclib and palbociclib subcohorts)

*n* (%)	Patients (*N*)	Events (*n*)	Median (months)	95% CIs (months)
Abemaciclib cohort^a^				
Duration of first abemaciclib therapy^b^	2834	1728	9.3	8.7, 10.0
Duration of first abemaciclib therapy by prior palbociclib use^a^				
No prior palbociclib use	1979 (69.8)	1193	10.5	9.5, 11.4
Prior palbociclib use	855 (30.2)	535	7.3	6.8, 8.3
Regimens of first abemaciclib therapy^c^				
ABE, FUL	1672 (59.0)			
ABE, LET or ANA	986 (34.8)			
ABE, EXE	73 (2.6)			
Abemaciclib monotherapy	58 (2.1)			
Palbociclib cohort^a^				
Duration of first palbociclib therapy^b^	4681	3396	8.7	8.1, 9.0
Duration of first palbociclib therapy by prior abemaciclib use^a^				
No prior abemaciclib use	4463 (95.3)	3280	8.8	8.3, 9.0
Prior abemaciclib use	218 (4.7)	116	7.0	5.8, 10.0
Regimens of first palbociclib therapy^c^				
PAL, FUL	2711 (57.9)			
PAL, LET or ANA	1763 (37.7)			
PAL, EXE	141 (3.0)			

^a^Patients who had any prescription of abemaciclib or palbociclib during follow-up were assigned to the “abemaciclib” or “palbociclib” subcohort, respectively, and could be included in both subcohorts if they were subsequently prescribed another CDK4 and 6 inhibitor during the follow-up period

^b^Duration is the period for which abemaciclib or palbociclib was prescribed. Patients were considered to be continuing the therapy and were censored at the last administration date of the therapy if there were ≤ 90 days between the end of the therapy and the end of data without prescription of another CDK4 and 6 inhibitor

^c^Regimens with a frequency > 1% shown

*ABE* abemaciclib, *ANA* anastrozole, *CDK* cyclin-dependent kinase, *CI* confidence interval, *EXE* exemestane, *FUL* fulvestrant, *LET* letrozole, *PAL* palbociclib

In both the abemaciclib and palbociclib subcohorts, 23.3% of patients started treatment on a reduced initial dose relative to the standard dose on the label. Use of a lower-than-recommended starting dose was more common among those aged ≥65 years (30.9%–32.6%) than those aged <65 years (15.5%–16.1%) and among those below median weight (25.2%–30.4%) than among those at or above median weight (20.7%–20.2%) but did not differ between BMI <18.5 and ≥18.5 kg/m^2^ categories (Table 6). The proportion of patients who experienced dose reduction during treatment was numerically higher in the palbociclib subcohort (69.3%) compared with the abemaciclib subcohort (53.0%), and the MPR was numerically higher in the abemaciclib subcohort compared with the palbociclib subcohort (median MPR: 0.96 and 0.91, respectively; Table 6).

**Table 6:** Initial dose, dose reduction, and medication possession ratio for first abemaciclib/palbociclib therapy (abemaciclib and palbociclib subcohorts)

	Abemaciclib	Palbociclib
Initial dose, mg/day		
Number of patients	2834	4681
Mean (SD)	273.9 (55.0)	118.6 (40.8)
Median	300	125
Proportion of patients whose initial dose was lower than the standard dose, *n* (%)^a^	661 (23.3)	1090 (23.3)
By age, *n*/*Nx* (%)^b^		
< 65 years	238/1538 (15.5)	386/2400 (16.1)
≥ 65 years	423/1296 (32.6)	704/2281 (30.9)
By weight, *n*/*Nx* (%)^b^		
< median kg^c^	90/357 (25.2)	177/582 (30.4)
≥ median kg^c^	75/362 (20.7)	118/585 (20.2)
By BMI, *n*/*Nx* (%)^b^		
< 18.5 kg/m^2^	142/631 (22.5)	253/1025 (24.7)
≥ 18.5 kg/m^2^	22/85 (25.9)	42/138 (30.4)
Dose reduction^d^		
Number of eligible patients	1380	2964
Patients with dose reduction, *n* (%)	732 (53.0)	2053 (69.3)
Medication possession ratio^d,e,f^		
Number of eligible patients	1380	2964
Mean (SD)	0.89 (0.16)	0.87 (0.14)
Median	0.96	0.91
Minimum–Maximum	0.02–1.0	0.06–1.0

*n* (%) shown, where % was calculated using the number of patients in the subcohorts as the denominator, unless otherwise indicated below

^a^Standard dose is 300 mg/day for abemaciclib and 125 mg/day for palbociclib

^b^*n* (%) shown where % was calculated using *Nx*

^c^Median bodyweight for each subcohort was used to define cutoff (abemaciclib, 54.1 kg; palbociclib, 53.0 kg)

^d^For dose reduction and MPR data, only patients who continued treatment for ≥ 30 days and then discontinued abemaciclib/palbociclib were eligible for the analysis

^e^Calculation of MPR = (total days supplied of CDK4 and 6 inhibitor) / (duration of CDK4 and 6 inhibitor therapy period) * 100 wherein total days supplied was obtained by summing up “days supplied” of all the prescriptions during the abemaciclib/palbociclib therapy period. If MPR exceeded 100%, MPR was defined as 100%

^f^MPR was adjusted for palbociclib based on dose schedule (3 weeks on, 1 week off)

*BMI* body mass index, *CDK* cyclin-dependent kinase, *MPR* medication possession ratio, *Nx* number of patients with data available, *SD* standard deviation

Diarrhea AEs of interest were more frequent in the abemaciclib subcohort (9.8%) compared with the palbociclib subcohort (1.5%), whereas neutropenia events that required G-CSF drugs were more common in the palbociclib subcohort (5.1%) compared to the abemaciclib subcohort (3.0%; Table 7). Use of the concomitant medication examined herein was generally similar between the subcohorts (Online Resource 8) except a numerically higher proportion of the abemaciclib subcohort was prescribed concomitant antidiarrheal agents (92.3%), antiemetic agents (41.5%), and probiotics (57.8%) compared with the palbociclib subcohort (7.9%, 22.9%, and 10.5%, respectively). Clinical monitoring tests between the two subcohorts were generally similar (Online Resource 8) except a numerically higher proportion of the abemaciclib subcohort underwent simple radiography (49.5%) compared with the palbociclib subcohort (38.5%).

**Table 7:** Incidence of adverse events of interest during first abemaciclib/palbociclib therapy (abemaciclib and palbociclib subcohorts)

AEs of interest^a^, *n* (%)	Abemaciclib *N* = 1380	Palbociclib *N* = 2964
Neutropenia^b^		
Number of eligible patients	1357 (98.3)	2943 (99.3)
Patients with AEs of interest	41 (3.0)	151 (5.1)
VTE^c^		
Number of eligible patients	1353 (98.0)	2907 (98.1)
Patients with AEs of interest	9 (0.7)	21 (0.7)
Liver disease^d^		
Number of eligible patients	1292 (93.6)	2822 (95.2)
Patients with AEs of interest	65 (5.0)	67 (2.4)
Diarrhea^e^		
Number of eligible patients	1372 (99.4)	2938 (99.1)
Patients with AEs of interest	135 (9.8)	43 (1.5)

^a^Only patients who continued treatment for ≥ 30 days and then discontinued abemaciclib/palbociclib were eligible for this analysis. Data shown include only AEs of interest that occurred during the index palbociclib/abemaciclib treatment period but not during a specified baseline period prior to the index date for each AE (30 days for diarrhea and neutropenia, 60 days for liver disease, and 365 days for VTE)

^b^Neutropenia events of interest were based on a diagnosis of neutropenia plus the prescription of ≥1 G-CSF drugs (pegfilgrastim, filgrastim, nartograstim, lenograstim) in the same claim month that overlaps with the period of measurement

^c^VTEs of interest were based on a diagnosis of VTE (DVT or PE) and any of the following within ± 1 claim months: (1) prescription of heparin, unfractionated heparin, or low-molecular-weight heparins; (2) prescription of fondaparinux; (3) inferior vena cava filter placement; (4) thrombus removal; (5) prescription of urokinase; (6) prescription of tissue plasminogen activator; and (7) prescription of warfarin or direct oral anticoagulant[29]

^d^Liver disease events of interest were based on a diagnosis of liver disease or the prescription of liver protection drugs (ursodeoxycholic acid or glycyrrhizin) and were excluded if liver cancer or liver metastasis was detected within ± 1 month of the claimed event

^e^Diarrhea events of interest were based on a diagnosis of diarrhea and of dehydration or abnormal electrolyte levels in the same claim month

*AE* adverse event, *DVT* deep vein thrombosis, *G-CSF* granulocyte colony stimulating factor, *PE* pulmonary embolism, *VTE* venous thromboembolism

## Discussion

This study included patients in Japan with HR+, HER2− breast cancer across a wide age range. The majority had claims records of metastatic disease and received ET prior to their first use of a CDK4 and 6 inhibitor. In recent years, abemaciclib and palbociclib were both frequently prescribed as the first CDK4 and 6 inhibitor therapy in Japanese patients, most commonly in combination with fulvestrant, which may indicate resistance to aromatase inhibitors in this cohort. After discontinuation of the first CDK4 and 6 inhibitor therapy, the most common first subsequent therapy was CDK4 and 6 inhibitor rechallenge, changing the CDK4 and 6 inhibitor or both the CDK4 and 6 inhibitor and ET in the regimen.

Although claims databases do not contain data on treatment effectiveness, the current study nevertheless offers some insights into the outcomes of real-world patterns of CDK4 and 6 inhibitor use based on TTD of treatments, as associations between TTD and survival endpoints have shown moderate to good correlation [30–32]. Median TTD for all breast cancer drugs after starting a CDK4 and 6 inhibitor, assumed to correspond to the period up to implementation of best supportive care, was 36.0 months in the CDK4/6i cohort. In comparison, clinical trials reported a median OS of 46.7 months for abemaciclib/fulvestrant [16] and 34.8 months for palbociclib/fulvestrant [13]. In addition, data from the phase 3 MONARCH 2 clinical trial showed trends in CFS indicating abemaciclib/fulvestrant delayed the need for subsequent chemotherapy compared to placebo/fulvestrant (median CFS, intent-to-treat population: 25.5 months [16]), on par with the median CFS following CDK4 and 6 inhibitor therapy observed in the current study (26.1 months). The median TTD for the first CDK4 and 6 inhibitor therapy in our study was longer for patients who were categorized as receiving it in relatively early lines of therapy (9.7 versus 12.0 months). This TTD was comparable with the previous PFS results from real-world studies of these agents although the real-world data are still sparce and further studies are warranted [33].

Of patients initiating another therapy after the first CDK4 and 6 therapy, 19.9% of patients initiated a different CDK4 and 6 inhibitor in combination with the same or different ET and 10.8% initiated CDK4 and 6 inhibitor/ET combination therapy wherein only the ET changed. This frequency of rechallenging with CDK4 and 6 inhibitor/ET therapy is of particular interest. Scant information exists in the literature regarding the efficacy and safety of specific CDK4 and 6 inhibitor/ET rechallenge treatment strategies, and the optimal CDK4 and 6 inhibitor/ET sequence is unknown. For patients prescribed a subsequent therapy, median TTD was numerically longest for patients who switched to a different CDK4 and 6 inhibitor compared to all other regimens. Although further investigation is necessary, this suggests that continuing CDK4 and 6 inhibition may have clinical benefit for patients with metastatic breast cancer.

Median TTD was 9.3 months for the abemaciclib subcohort and 8.7 months for the palbociclib subcohort. In both subcohorts, the median TTD was numerically longer in the patients who received CDK4 and 6 inhibitor for the first time than in those who were previously treated with another CDK4 and 6 inhibitor (10.5 versus 7.3 months for abemaciclib; 8.8 versus 7.0 months for palbociclib). In both subcohorts, 23.3% of the patients started at a lower-than-recommended dose of CDK4 and 6 inhibitor, and this proportion was higher in older patients. The finding may indicate physicians tend to use lower doses in management of elderly patients, possibly in consideration of the more common comorbidity and deteriorated health status associated with age, although patients’ values and preferences are also suggested to be important in therapy decisions [34].

The incidence of AEs of interest and use of concomitant therapies during therapies with CDK4 and 6 inhibitors were generally consistent with clinical study observations for palbociclib and abemaciclib [6, 7, 9, 11, 13, 16, 17, 20, 23, 35, 36]. In particular, the higher incidence of diarrhea AEs of interest and higher use of antidiarrhea agents observed in the abemaciclib subcohort are consistent with MONARCH 2 and MONARCH 3 findings and the main management strategy in these studies [35]. Similarly, the higher incidence of neutropenia observed with palbociclib is consistent with previously reported clinical data [37, 38] although only neutropenia events requiring prescription of G-CSF drugs were captured herein, per the definition of neutropenia used in this study.

This study had limitations. Notably, this was a descriptive analysis by design, and results were not adjusted for baseline differences between groups to allow for statistical comparisons between treatments. In addition, the MDV database has several limitations on available data that affected the current analyses. Although the CDK4/6i cohort was defined as the patients who were prescribed the therapy for the first time, this cohort may have included those who started the CDK 4 and 6 inhibitor therapy as a later line of therapy because the true first-line therapy for advanced breast cancer treatment could not be reliably identified from the claims records. In addition, the database cannot track a patient across multiple hospitals, so patients may have been lost or counted multiple times if they received treatment at more than one hospital and the full 5-year treatment history period was not available for all patients. Additionally, reasons underlying treatment discontinuation are not available, so it is not possible to discern between patients discontinuing treatment due to progressive disease, AEs, or other reasons. Finally, some important clinical information essential for treatment choice in clinical practice (e.g., cancer stage, performance status) and common effectiveness endpoints (e.g., OS, PFS, tumor response) were not available or were largely missing from the database.

## Conclusion

This study extends our understanding of real-world CDK4 and 6 inhibitor use in Japan, providing insight into current treatment practices for HR+, HER2− advanced breast cancer, specifically in relation to treatment patterns of CDK4 and 6 inhibitor use and their clinical outcomes. These data may inform future treatment strategies, including optimization of treatment sequence.

## Supplementary Information

Below is the link to the electronic supplementary material.

Supplementary file1 (PDF 340 KB)

## Data Availability

The data that support the findings of this study were from the Medical Data Vision Co., Ltd. Restrictions apply to the availability of these data, which were used under license to Eli Lilly Japan K.K. for the current study, and so are not publicly available. The data are however available from the authors upon reasonable request and with permission of the Medical Data Vision Co., Ltd.

## References

[1] International Agency for Research on Cancer (2020) Cancer Today. Japan Fact Sheet—Globocan 2020, available at https://gco.iarc.fr/today/data/factsheets/populations/392-japan-fact-sheets.pdf. Accessed 03 May 2022.

[2] National Cancer Institute (2022) Cancer stat facts: Female breast cancer subtypes. https://seer.cancer.gov/statfacts/html/breast-subtypes.html. Accessed 10 April 2022.

[3] Spring LM, Wander SA, Andre F, Moy B, Turner NC, Bardia A (2020). Cyclin-dependent kinase 4 and 6 inhibitors for hormone receptor-positive breast cancer: past, present, and future. Lancet.

[4] Finn RS, Crown JP, Ettl J, Schmidt M, Bondarenko IM, Lang I (2016). Efficacy and safety of palbociclib in combination with letrozole as first-line treatment of ER-positive, HER2-negative, advanced breast cancer: expanded analyses of subgroups from the randomized pivotal trial PALOMA-1/TRIO-18. Breast Cancer Res.

[5] Finn RS, Crown JP, Lang I, Boer K, Bondarenko IM, Kulyk SO (2015). The cyclin-dependent kinase 4/6 inhibitor palbociclib in combination with letrozole versus letrozole alone as first-line treatment of oestrogen receptor-positive, HER2-negative, advanced breast cancer (PALOMA-1/TRIO-18): a randomised phase 2 study. Lancet Oncol.

[6] Goetz MP, Toi M, Campone M, Sohn J, Paluch-Shimon S, Huober J (2017). MONARCH 3: abemaciclib as initial therapy for advanced breast cancer. J Clin Oncol.

[7] Johnston S, Martin M, Di Leo A, Im SA, Awada A, Forrester T (2019). MONARCH 3 final PFS: a randomized study of abemaciclib as initial therapy for advanced breast cancer. NPJ Breast Cancer.

[8] O'Shaughnessy J, Petrakova K, Sonke GS, Conte P, Arteaga CL, Cameron DA (2018). Ribociclib plus letrozole versus letrozole alone in patients with de novo HR+, HER2– advanced breast cancer in the randomized MONALEESA-2 trial. Breast Cancer Res Treat.

[9] Rugo HS, Finn RS, Diéras V, Ettl J, Lipatov O, Joy AA (2019). Palbociclib plus letrozole as first-line therapy in estrogen receptor-positive/human epidermal growth factor receptor 2-negative advanced breast cancer with extended follow-up. Breast Cancer Res Treat.

[10] Slamon DJ, Neven P, Chia S, Fasching PA, De Laurentiis M, Im SA (2018). Phase III randomized study of ribociclib and fulvestrant in hormone receptor-positive, human epidermal growth factor receptor 2-negative advanced breast cancer: MONALEESA-3. J Clin Oncol.

[11] Sledge GW, Toi M, Neven P, Sohn J, Inoue K, Pivot X (2017). MONARCH 2: abemaciclib in combination with fulvestrant in women with HR+/HER2- advanced breast cancer who had progressed while receiving endocrine therapy. J Clin Oncol.

[12] Turner NC, Ro J, André F, Loi S, Verma S, Iwata H (2015). Palbociclib in hormone-receptor-positive advanced breast cancer. N Engl J Med.

[13] Cristofanilli M, Rugo HS, Im SA, Slamon DJ, Harbeck N, Bondarenko I (2022). Overall survival with palbociclib and fulvestrant in women with HR+/HER2– ABC: updated exploratory analyses from PALOMA-3, a double-blind, phase 3 randomized study. Clin Cancer Res.

[14] Hortobagyi GN, Stemmer SM, Burris HA, Yap YS, Sonke GS, Hart L (2022). Overall survival with ribociclib plus letrozole in advanced breast cancer. N Engl J Med.

[15] Slamon DJ, Neven P, Chia S, Fasching PA, De Laurentiis M, Im SA (2020). Overall survival with ribociclib plus fulvestrant in advanced breast cancer. N Engl J Med.

[16] Sledge GW, Toi M, Neven P, Sohn J, Inoue K, Pivot X (2020). The effect of abemaciclib plus fulvestrant on overall survival in hormone receptor-positive, ERBB2-negative breast cancer that progressed on endocrine therapy-MONARCH 2: a randomized clinical trial. JAMA Oncol.

[17] Turner NC, Slamon DJ, Ro J, Bondarenko I, Im SA, Masuda N (2018). Overall survival with palbociclib and fulvestrant in advanced breast cancer. N Engl J Med.

[18] PMDA (2017) Pharmaceutical and Medical Devices Agency. New drugs approved in September 2017 and 2018. https://www.pmda.go.jp/english/review-services/reviews/approved-information/drugs/0002.html. Accessed 10 April 2022.

[19] Shimoi T, Nagai SE, Yoshinami T, Takahashi M, Arioka H, Ishihara M (2020). The Japanese Breast Cancer Society clinical practice guidelines for systemic treatment of breast cancer, 2018 edition. Breast Cancer.

[20] Inoue K, Masuda N, Iwata H, Takahashi M, Ito Y, Miyoshi Y (2021). Japanese subpopulation analysis of MONARCH 2: phase 3 study of abemaciclib plus fulvestrant for treatment of hormone receptor-positive, human epidermal growth factor receptor 2-negative breast cancer that progressed on endocrine therapy. Breast Cancer.

[21] Masuda N, Inoue K, Nakamura R, Rai Y, Mukai H, Ohno S (2019). Palbociclib in combination with fulvestrant in patients with hormone receptor-positive, human epidermal growth factor receptor 2-negative advanced breast cancer: PALOMA-3 subgroup analysis of Japanese patients. Int J Clin Oncol.

[22] Mukai H, Shimizu C, Masuda N, Ohtani S, Ohno S, Takahashi M (2019). Palbociclib in combination with letrozole in patients with estrogen receptor-positive, human epidermal growth factor receptor 2-negative advanced breast cancer: PALOMA-2 subgroup analysis of Japanese patients. Int J Clin Oncol.

[23] Takahashi M, Tokunaga E, Mori J, Tanizawa Y, van der Walt JS, Kawaguchi T (2022). Japanese subgroup analysis of the phase 3 MONARCH 3 study of abemaciclib as initial therapy for patients with hormone receptor-positive, human epidermal growth factor receptor 2-negative advanced breast cancer. Breast Cancer.

[24] Nakamura M (2016). Utilization of MDV data and data quality control. Jap J Pharmacoepidemiol.

[25] Medical Data Vision (2022) https://en.mdv.co.jp/about-mdv-database/ Accessed 02 May 2022.

[26] Sone S (2015). Ethical guidelines for clinical trials in medical research involving human subjects. Gan To Kagaku Ryoho.

[27] World Health Organisation (2022) International Statistical Classification of Diseases and Related Health Problems, 10th Revision [ICD-10]. https://www.who.int/standards/classifications/classification-of-diseases. Accessed 03 May 2022.

[28] Wade DT, Collin C (1988). The Barthel ADL Index: a standard measure of physical disability?. Int Disabil Stud.

[29] Fujiya M, Kawaguchi T, Arai S, Isogawa N, Hiro S, Matsumoto F (2022). Real-world insurance claims analysis of venous thromboembolism in Japanese patients with inflammatory bowel disease. Dig Dis Sci.

[30] Blumenthal GM, Gong Y, Kehl K, Mishra-Kalyani P, Goldberg KB, Khozin S (2019). Analysis of time-to-treatment discontinuation of targeted therapy, immunotherapy, and chemotherapy in clinical trials of patients with non-small-cell lung cancer. Ann Oncol.

[31] Stewart M, Norden AD, Dreyer N, Henk HJ, Abernethy AP, Chrischilles E (2019). An exploratory analysis of real-world end points for assessing outcomes among immunotherapy-treated patients with advanced non-small-cell lung cancer. JCO Clin Cancer Inform.

[32] Yang G, Ma D, Xu H, Yang L, Li J, Xing P (2019). Treatment duration as a surrogate endpoint to evaluate the efficacy of crizotinib in sequential therapy for patients with advanced ALK-positive non-small cell lung cancer: a retrospective, real-world study. Cancer Med.

[33] Harbeck N, Bartlett M, Spurden D, Hooper B, Zhan L, Rosta E (2021). CDK4/6 inhibitors in HR+/HER2- advanced/metastatic breast cancer: a systematic literature review of real-world evidence studies. Future Oncol.

[34] Jolly T, Williams GR, Jones E, Muss HB (2012). Treatment of metastatic breast cancer in women aged 65 years and older. Womens Health (Lond).

[35] Masuda N, Chen Y, Kawaguchi T, Dozono K, Toi M (2022). Safety in Japanese advanced breast cancer patients who received abemaciclib in MONARCH 2 and MONARCH 3: assessment of treatment-emergent neutropenia, diarrhea, and increased alanine aminotransferase and aspartate aminotransferase levels. Cancer Manag Res.

[36] Masuda N, Kosaka N, Iwata H, Toi M (2021). Palbociclib as an early-line treatment for Japanese patients with hormone receptor-positive/human epidermal growth factor receptor 2-negative advanced breast cancer: a review of clinical trial and real-world data. Int J Clin Oncol.

[37] Diéras V, Rugo HS, Schnell P, Gelmon K, Cristofanilli M, Loi S (2019). Long-term pooled safety analysis of palbociclib in combination with endocrine therapy for HR+/HER2– advanced breast cancer. J Natl Cancer Inst.

[38] Ettl J, Im SA, Ro J, Masuda N, Colleoni M, Schnell P (2020). Hematologic adverse events following palbociclib dose reduction in patients with hormone receptor-positive/human epidermal growth factor receptor 2-negative advanced breast cancer: pooled analysis from randomized phase 2 and 3 studies. Breast Cancer Res.

