# Supplementary material for: Patient characteristics, treatment patterns, and outcomes of hormone receptor-positive, human epidermal growth factor receptor 2-negative advanced breast cancer patients prescribed cyclin-dependent kinase 4 and 6 inhibitors: large-scale data analysis using a Japanese claims database
Source: Breast Cancer Res Treat. 2022 Nov 21;197(2):435–47. doi: 10.1007/s10549-022-06816-9 (PMC9823084; doi:10.1007/s10549-022-06816-9)
Supplement: Supplementary file 1 — Supplementary file1 (PDF 340 KB) [file 10549_2022_6816_MOESM1_ESM.pdf]

Supplementary information – Online resources

Patient characteristics, treatment patterns, and outcomes of hormone receptor-positive, human epidermal growth factor receptor 2-negative advanced breast cancer patients prescribed cyclin-dependent kinase 4 and 6 inhibitors: large-scale data analysis using a Japanese claims database

Masaaki Kawai[1], Masahiro Takada[2], Takahiro Nakayama[3], Norikazu Masuda[4], Hirokazu Shiheido[5], Zhihong Cai[5], Yu-Jing Huang[6], Tsutomu Kawaguchi[5], Yoshinori Tanizawa[5]

[1]Department of Surgery I, Yamagata University Graduate School of Medical Science, Yamagata, Japan; [2]Department of Breast Surgery, Kyoto University Graduate School of Medicine, Kyoto, Japan; [3]Department of Breast and Endocrine Surgery, Osaka International Cancer Institute, Osaka, Japan; [4]Department of Breast and Endocrine Surgery, Nagoya University Graduate School of Medicine, Nagoya, Japan; [5]Japan Drug Development and Medical Affairs, Eli Lilly Japan K.K., Kobe, Japan; [6]Global Patient Safety, Eli Lilly and Company, Indianapolis, Indiana, USA

Correspondence: Yoshinori Tanizawa, tanizawa_yoshinori@lilly.com

Online Resource 1. List of breast cancer drugs for treatment pattern analysis

Class	Drugs
**Alkylating agents[a]**	Cyclophosphamide
**Antimetabolites[a]**	Capecitabine
	Doxifluridine
	Fluorouracil
	Gemcitabine
	Methotrexate
	Tegafur/uracil
	Tegafur/gimeracil/oteracil (S-1)
**Plant alkaloids and other natural products[a]**	Docetaxel
	Irinotecan
	nab-paclitaxel
	Paclitaxel
	Vinorelbine
**Cytotoxic antibiotics and related substances[a]**	Doxorubicin
	Epirubicin
	Mitomycin C
	Mitoxantrone
**Platinum agents[a]**	Carboplatin
	Cisplatin
**Other antineoplastic agents**	Bevacizumab[a]
	Eribulin[a]
	Everolimus
	Olaparib[a]
**Anticancer drugs – CDK4 and 6 inhibitors**	Abemaciclib
	Palbociclib

**Anticancer drugs – anti-HER2 drugs**[b]	Lapatinib
	Pertuzumab
	Trastuzumab
	Trastuzumab deruxtecan
	Trastuzumab emtansine
**Endocrine therapies**[c]	Anastrozole
	Exemestane
	Fulvestrant
	Letrozole
	Medroxyprogesterone
	Tamoxifen
	Toremifene

[a]Included in the CFS analysis. For the intravenous CFS analysis, drugs administered orally were excluded (i.e., capecitabine, doxifluridine, olaparib, tegafur/gimeracil/oteracil, and tegafur/uracil).
[b]Used as a surrogate for "HER2+". Patients in this category were removed to define the "HER2-" population.
[c]Used as a surrogate for "HR+".

CDK = cyclin-dependent kinase; CFS = chemotherapy-free survival; HER2+ = human epidermal growth factor receptor 2-positive; HER2- = human epidermal growth factor receptor 2-negative; HR+ = hormone-receptor-positive; nab = nanoparticle-albumin-bound

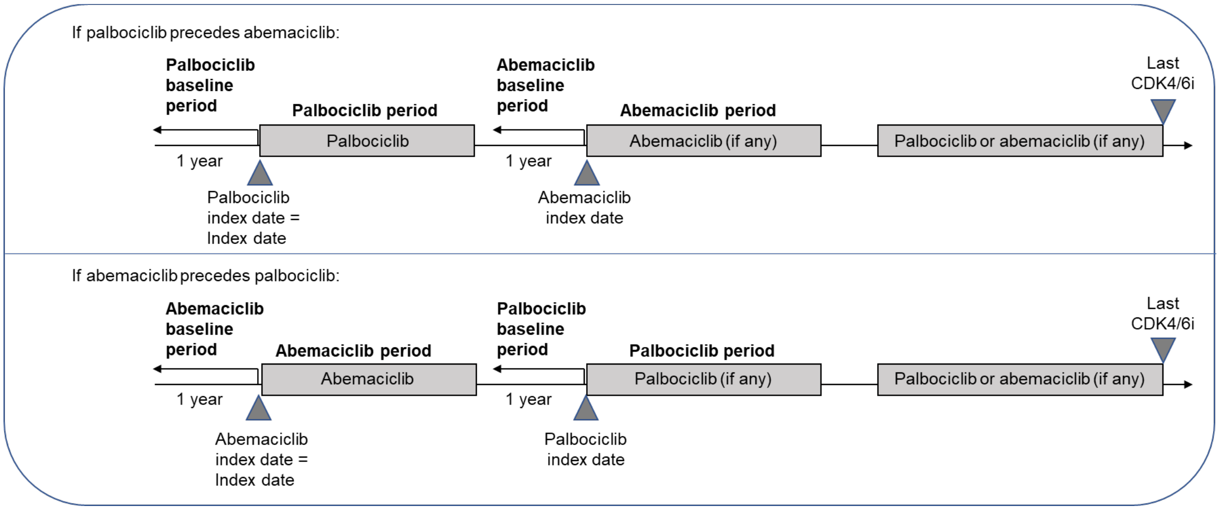

Online Resource 2. Data analysis periods for subcohorts. Schematic depicting data analysis periods for the subcohorts, inclusive of potential multiple episodes of CDK4 and 6 inhibitor therapies. Data analysis periods ("palbociclib period" or "abemaciclib period") started at the first prescription of any CDK4 and 6 inhibitor (the index date) and ended on the last projected dose date (last prescription date plus the number of days of drug supply – 1) for that CDK4 and 6 inhibitor. If more than one CDK4 and 6 inhibitor was used, periods were defined for each, starting on the index date for the first period of CDK4 and 6 inhibitor use and extending to the last projected dose date or one day before the start of the subsequent CDK4 and 6 inhibitor, whichever occurred first.

CDK = cyclin-dependent kinase; CDK4/6i = CDK4 and 6 inhibitor.

Online Resource 3. Age distribution of patients

Age at index date, years, n (%)	CDK4/6i cohort N=6442
25-29	4 (0.1)
30-34	25 (0.4)
35-39	105 (1.6)
40-44	295 (4.6)
45-49	579 (9.0)
50-54	733 (11.4)
55-59	761 (11.8)
60-64	855 (13.3)
65-69	1085 (16.8)
70-74	1013 (15.7)
75-79	576 (8.9)
80-84	288 (4.5)
85-89	96 (1.5)
90-	27 (0.4)

CDK4/6i = cyclin-dependent kinase 4 and 6 inhibitor

Online Resource 4. Treatment history up to 5 years prior to the index date

Treatment, n (%)	CDK4/6i cohort N=6442
**Total**	6442 (100)
**Any radiotherapy**	1303 (20.2)
LINAC	1293 (20.1)
Electromagnetic thermal therapy	6 (0.1)
Gamma knife	12 (0.2)
Particle beam	0
SAVI	0
Whole-body radiation	0
**Any endocrine therapy**	4970 (77.2)
Fulvestrant	2395 (37.2)
Letrozole	2066 (32.1)
Tamoxifen	1575 (24.5)
Anastrozole	1351 (21.0)
Exemestane	985 (15.3)
Toremifene	488 (7.6)
Medroxyprogesterone acetate	202 (3.1)
**Any anticancer drug**	2619 (40.7)
Cyclophosphamide	1226 (19.0)
Paclitaxel	1009 (15.7)
Bevacizumab	775 (12.0)
Epirubicin	750 (11.6)
Eribulin	626 (9.7)
Tegafur/gimeracil/oteracil	607 (9.4)
Docetaxel	588 (9.1)
Capecitabine	549 (8.5)
Everolimus	435 (6.8)
Fluorouracil	376 (5.8)
Doxorubicin	180 (2.8)
Vinorelbine	141 (2.2)
nabPTX	132 (2.1)
Gemcitabine	116 (1.8)
Tegafur/uracil	76 (1.2)
Doxifluridine	42 (0.7)
Methotrexate	23 (0.4)
Irinotecan	13 (0.2)
Olaparib	12 (0.2)
Carboplatin	11 (0.2)
Cisplatin	4 (0.1)
Mitoxantrone	1 (0.0)

CDK4/6i = cyclin-dependent kinase 4 and 6 inhibitor; LINAC = linear accelerator; nabPTX = nanoparticle-albumin-bound paclitaxel; SAVI = strut-adjusted volume implant.

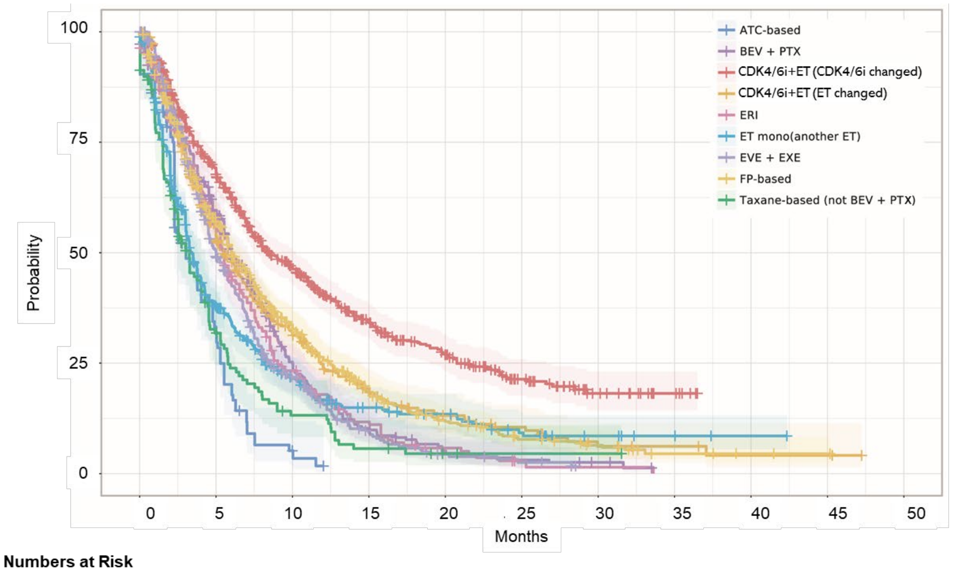

Time to discontinuation

First subsequent therapy	n	Events	Median (months)	95% CIs
ATC-based	94	84	3.0	2.3, 4.8
BEV+PTX	359	290	6.0	5.5, 7.0
CDK4/6i+ET (CDK4/6i changed)	748	464	8.5	7.6, 10.3
CDK4/6i+ET (ET changed)	407	296	5.5	5.0, 6.6
ERI	272	229	5.5	5.0, 6.3
ET monotherapy (another ET)	432	330	3.4	3.1, 3.8
EVE+EXE	431	344	5.0	4.5, 5.8
FP-based	659	506	6.0	5.6, 7.0
Taxane-based (not BEV+PTX)	138	118	3.3	2.5, 4.3

Numbers at Risk

Time (months)	0	5	10	15	20	25	30	35	40	45	50
ATC-based	94	28	4	0	0	0	0	0	0	0	0
BEV+PTX	359	187	65	25	10	6	3	0	0	0	0
CDK4/6i+ET (CDK4/6i changed)	748	465	251	137	89	43	19	6	0	0	0
CDK4/6i+ET (ET changed)	407	193	91	40	22	14	6	4	2	2	0
ERI	272	140	50	20	8	2	1	0	0	0	0
ET monotherapy (another ET)	432	136	59	32	25	13	6	3	1	0	0
EVE+EXE	431	188	67	22	8	4	0	0	0	0	0
FP-based	659	341	161	76	40	20	13	4	2	1	0
Taxane-based (not BEV+PTX)	138	37	14	6	2	1	1	0	0	0	0

Online Resource 5. Time to discontinuation of the first subsequent therapy (CDK4/6i cohort). Kaplan Meier plots of TTD of the first therapy prescribed after the first CDK4 and 6 inhibitor. The “CDK4/6i + ET (CDK4/6i changed)” category includes patients prescribed CDK4 and 6 inhibitor/ET combination regimens who subsequently either initiated a different CDK4 and 6 inhibitor with the same ET or both a different CDK4 and 6 inhibitor and a different ET.

ATC = anthracycline; BEV = bevacizumab; CDK = cyclin-dependent kinase; CDK4/6i = CDK4 and 6 inhibitor; CI = confidence interval; ERI = eribulin; ET = endocrine therapy; EVE = everolimus; EXE = exemestane; FP = fluoropyrimidine; PTX = paclitaxel; TTD = time to discontinuation

Online Resource 6. First subsequent therapy regimens after removal of patients who were prescribed prior metastatic breast cancer drugs except for fulvestrant[a]

First subsequent therapy, n (%)	CDK4/6i cohort N=4523
Patients who initiated a subsequent therapy	2453 (54.2)
CDK4 and 6 inhibitor + ET (CDK4 and 6 inhibitor changed)[b]	523 (21.3)
FP-based	457 (18.6)
EVE + EXE	311 (12.7)
ET mono (another ET)	310 (12.6)
CDK4 and 6 inhibitor + ET (ET changed)	272 (11.1)
BEV + PTX	220 (9.0)
ERI	146 (6.0)
Others	89 (3.6)
Taxane-based (not BEV + PTX)	70 (2.9)
ATC-based	55 (2.2)

n (%) shown.

[a]Metastatic breast cancer drugs included capecitabine, gemcitabine, S-1, irinotecan, vinorelbine, nab-paclitaxel, bevacizumab, eribulin, everolimus, palbociclib, abemaciclib, and olaparib. Fulvestrant is indicated for metastatic breast cancer but was not removed in this analysis because it can be used in earlier lines of therapy.

[b]Category includes patients who were prescribed CDK 4 and 6 inhibitor/ET combination regimens who subsequently either initiated a different CDK4 and 6 inhibitor with the same ET or both a different CDK4 and 6 inhibitor and a different ET.

ATC = anthracycline; BEV = bevacizumab; CDK = cyclin-dependent kinase; CDK4/6i = CDK4 and 6 inhibitor; ERI = eribulin; ET = endocrine therapy; EVE = everolimus; EXE = exemestane; FP = fluoropyrimidine; PTX = paclitaxel.

Online Resource 7. Time to discontinuation of therapy and chemotherapy-free survival in the CDK4/6i cohort after removal of patients who were prescribed prior metastatic breast cancer drugs except for fulvestrant[a]

Time-to-event measure	Patients (N)	Events (n)	Median (months)	95% CIs (months)
TTD, first CDK4 and 6 inhibitor therapy[b]	4523	2921	12.0	11.3, 12.7
TTD, first subsequent therapy[b]	2453	1734	6.1	5.8, 6.5
TTD, overall breast cancer drugs (from index date)[c]	4523	1296	NA	42.7, NA
CFS[d]	4261	1668	26.9	25.1, 28.6
Intravenous CFS[e]	4261	1321	36.5	34.2, 38.5

Treatment regimens were defined as the combination of breast cancer drugs that were prescribed within the first 21 days of each line of therapy. The line of therapy ended when the patient either: 1) terminated all the breast cancer drugs in the regimen (end date: date of last prescription plus the number of days of supply - 1 day); or 2) added a new breast cancer drug that was not included in the regimen (i.e., causing the treatment line to advance), whichever occurred first.

[a]Metastatic breast cancer drugs included capecitabine, gemcitabine, tegafur/gimeracil/oteracil (S-1), irinotecan, vinorelbine, nab-paclitaxel, bevacizumab, eribulin, everolimus, palbociclib, abemaciclib, and olaparib. Fulvestrant is indicated for metastatic breast cancer but was not removed in this analysis because it can be used in earlier lines of therapy.

[b]Patients were considered to be continuing the line and were censored at the last administration date of the line if there were ≤90 days between the end of the line and the end of data without a subsequent line of therapy.

[c]Patients with ≤90 days between the estimated last dose of breast cancer drugs and end of data were censored for therapy duration at the last dose, as such patients were likely to be on treatment at the last visit.

[d]Time from the CDK4 and 6 inhibitor index date to the date of first chemotherapy use or death. If no events occurred, patients were censored at the last hospital visit record. Only patients without chemotherapy in the 1-year baseline period were included in the analysis.

[e]Time from the CDK4 and 6 inhibitor index date to the date of first intravenous chemotherapy use or death. If no events occurred, patients were censored at the last hospital visit record. Only patients without chemotherapy in the 1-year baseline period were included in the analysis.

CDK = cyclin-dependent kinase; CDK4/6i = CDK4 and 6 inhibitor; CFS = chemotherapy-free survival; CI = confidence interval; MBC = metastatic breast cancer; NA = not available; TTD = time to discontinuation.

Online Resource 8. Concomitant therapy and monitoring tests during first CDK4 and 6 inhibitor therapy (abemaciclib and palbociclib subcohorts)

n (%)	Abemaciclib N=1380	Palbociclib N=2964
**Concomitant therapies**		
Pain killers	758 (54.9)	1621 (54.7)
Denosumab	599 (43.4)	1330 (44.9)
Bisphosphonate	107 (7.8)	257 (8.7)
Antidiarrheal agents	1273 (92.3)	235 (7.9)
Probiotics	797 (57.8)	312 (10.5)
Antiemetics	573 (41.5)	680 (22.9)
Liver protection drugs	114 (8.3)	148 (5.0)
Systemic steroids	162 (11.7)	230 (7.8)
Thrombolytic drugs	180 (13.0)	312 (10.5)
**Monitoring tests**		
Liver enzyme test	1376 (99.7)	2952 (99.6)
Peripheral blood test	1168 (84.6)	2632 (88.8)
Scintigram	153 (11.1)	302 (10.2)
SPECT	2 (0.1)	7 (0.2)
CT	947 (68.6)	1909 (64.4)
PET	16 (1.2)	19 (0.6)
PET-CT	58 (4.2)	165 (5.6)
MRI	250 (18.1)	548 (18.5)
PET-MRI	0	0
Mammography	19 (1.4)	58 (2.0)
Simple radiography	683 (49.5)	1141 (38.5)

Only patients who continued treatment for ≥30 days and then discontinued abemaciclib/palbociclib were eligible for this analysis.

CT = computed tomography; MRI = magnetic resonance imaging; PET = positron emission tomography; SPECT = single-photon emission computerized tomography.
